# Association of lower limb muscle mass and energy expenditure with visceral fat mass in healthy men

**DOI:** 10.1186/1758-5996-6-27

**Published:** 2014-02-26

**Authors:** Shusuke Yagi, Muneyuki Kadota, Ken-ichi Aihara, Koji Nishikawa, Tomoya Hara, Takayuki Ise, Yuka Ueda, Takashi Iwase, Masashi Akaike, Michio Shimabukuro, Shinsuke Katoh, Masataka Sata

**Affiliations:** 1 Department of Cardiovascular Medicine, The University of Tokushima Graduate School of Health Biosciences, 3-18-15 Kuramoto-cho, Tokushima 770-8503, Japan; 2 Department of Medicine and Bioregulatory Sciences, The University of Tokushima Graduate School of Health Biosciences, Tokushima, Japan; 3 Department of Rehabilitation, Tokushima University Hospital, Tokushima, Japan; 4 Department of Medical Education, The University of Tokushima Graduate School of Health Biosciences, Tokushima, Japan; 5 Department of Cardio-Diabetes Medicine, The University of Tokushima Graduate School of Health Biosciences, Tokushima, Japan

**Keywords:** Exercise, Skeletal muscle, Metabolic syndrome, Prevention

## Abstract

**Background:**

A high-calorie diet and physical inactivity, an imbalance between caloric intake and energy consumption, are major causes of metabolic syndrome (MetS), which manifests as accumulation of visceral fat and insulin resistance. However, the lifestyle-related factors associated with visceral fat mass in healthy men are not fully understood.

**Methods:**

We evaluated visceral fat area (VFA), skeletal muscle mass, caloric intake, and energy expenditure in 67 healthy male participants (mean age, 36.9 ± 8.8 years; body mass index 23.4 ± 2.5 kg/m^2^).

**Results:**

Multiple regression analysis showed that the total skeletal muscle mass (*P* < 0.001) were negatively and age (*P* < 0.001) were positively associated with VFA. Lower limb muscle mass (*P* < 0.001) was strongly associated with VFA. However, total caloric intake, total energy expenditure, and energy expenditure during exercise were not associated with VFA.

**Conclusions:**

Skeletal muscle mass especially lower limb muscle mass negatively contributes to visceral fat mass in healthy men. Therefore, maintaining lower limb muscular fitness through daily activity may be a useful strategy for controlling visceral obesity and metabolic syndrome.

## Introduction

The imbalance between caloric intake and energy consumption, high-calorie diets and physical inactivity, are major causes of metabolic syndrome (MetS), which manifests as accumulation of visceral fat and insulin resistance [1]. The prevention of MetS is an important issue, as it is a major cause of cardiovascular disease (CVD) [2,3].

Lifestyle intervention including caloric restriction and exercise is the preferred approach to reduce the incidence of MetS [4,5]. Exercise not only increases energy consumption but also improves muscle metabolism through increased glucose uptake in skeletal muscles [6]. In addition, exercise enhances skeletal muscle mass, suggesting that skeletal muscle mass could be a parameter of exercise duration and intensity. Exercise prevents visceral obesity [7]; however, the relashonship between viseral obesity and skeletal muscle mass remains unclear.

The American Heart Association therefore recommends weight reduction to a BMI of <25 kg/m^2^) with exercise duration of at least 30 min performed 5 times a week at moderate intensity [1]; however, it is unclear which exercise should be used and which skeletal muscles should be targeted to effectively reduce visceral fat mass in healthy subjects. Since the prevalence of MetS is increasing worldwide, healthy subjects are potentially at risk of MetS [8-10]. Therefore, it is important to identify the risk factors for visceral fat obesity in healthy subjects to prevent MetS. In order to clarify these issues, we evaluated visceral fat mass, skeletal muscle mass, caloric intake, and energy consumption in healthy Japanese men and identified lifestyle-related factors associated with visceral fat mass.

## Methods

We recruited 67 healthy male volunteers aged between 20 and 85 years (mean age, 36.9 ± 8.8 years; body mass index (BMI) 23.4 ± 2.5 kg/m^2^). Visceral fat area (VFA) and subcutaneous fat area (SFA) were measured using a fat area analyzer (Dual Scan HDS-2000®; Omron, Japan) [11,12]. Studies have shown that the correlation coefficient between VFA measured by the fat area analyzer and VFA measured by computed tomography was r = 0.88 (p < 0.001) [12]. The repeatability of the fat area analyzer was evaluated by the Bland–Altman plot, which has been described elsewhere [12]. These data indicate that this indirect measurement of VFA has a high correlation coefficient with VFA evaluated by computed tomography and does not involve X-ray exposure. Because VFA and SFA were compared with obesity-related variables, which were adjusted with body size represented by body surface area or body weight, VFA and SFA were indexed with body surface area (BSA) as visceral fat area index (VFAI) and subcutaneous fat area index (SFAI), respectively. Skeletal muscle mass was measured with a body composition analyzer (Inbody 7200®; Biospace, Korea) [13]. Body weight and waist circumstance were measured, and BMI was calculated as an index of obesity.

Energy expenditure and total caloric intake was calculated using a questionnaire for food and exercise frequency. Energy expenditure during exercise was defined as energy consumed during exercise per day. Total energy expenditure was defined as energy consumed for daily activity, which includes energy expenditure during exercise. Total caloric intake, energy expenditure during exercise, and total energy expenditure were assessed for 7 days. These values were then averaged per day [14,15].

The study protocol was approved by the Ethics Committee at the Tokushima University Hospital.

### Statistical analysis

For continuous variables, each value is expressed as the mean ± SD. Single regression analysis was used to assess the correlation between VFA and obesity-related parameters. The degree of association among independent variables, including VFAI, age, skeletal muscle mass, energy expenditure, caloric intake, and parts of skeletal muscles, was assessed by multiple regression analyses (stepwise regression model). All statistical analyses were performed using SPSS software. Statistical significance was defined as *P* < 0.05.

## Results

### Clinical characteristics of subjects

The clinical characteristics of the subjects are presented in Table 1.

**Table 1:** Clinical characteristics of subjects

**Variables (n = 67)**	**Mean ± SD**
Age (years)	36.9 ± 8.8
BW (kg)	69.2 ± 8.3
BMI (kg/m^2^)	23.4 ± 2.5
Waist circumference (cm^2^)	84.2 ± 7.4
VFA (cm^2^)	75.0 ± 29.5
VFAI (cm^2^)	41.0 ± 14.7
SFA (cm^2^)	160.3 ± 56.9
SFAI (cm^2^)	87.6 ± 27.7
Fat weight (kg)	15.1 ± 5.3
Fat weight/BW (%)	21.5 ± 5.6
Skeletal muscle weight	
Total body (kg)	30.4 ± 3.0
Total body/BW(%)	44.3 ± 0.03
Upper limbs, (kg)	5.8 ± 0.7
Upper limbs/BW, (%)	8.3 ± 0.7
Lower limbs (kg)	17.5 ± 1.9
Lower limbs/BW (%)	25.5 ± 2.3
Truncal muscle (kg)	7.1 ± 0.9
Truncal muscle/BW (%)	10.3 ± 1.3

BW, body weight; BMI, body mass index; VFA, visceral fat area; VFAI, visceral fat area index; SFA, subcutaneous fat area; SFAI, subcutaneous fat area index.

### VFAI is inversely associated with skeletal muscle mass and energy expenditure

**Figure 1:**
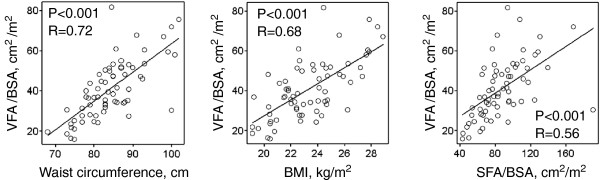
**Waist circumference, BMI, and SFA are associated with VFA.** VFA: visceral fat area, BMI: body mass index, SFA: subcutaneous fat area, BSA: body surface area.

**Figure 2:**
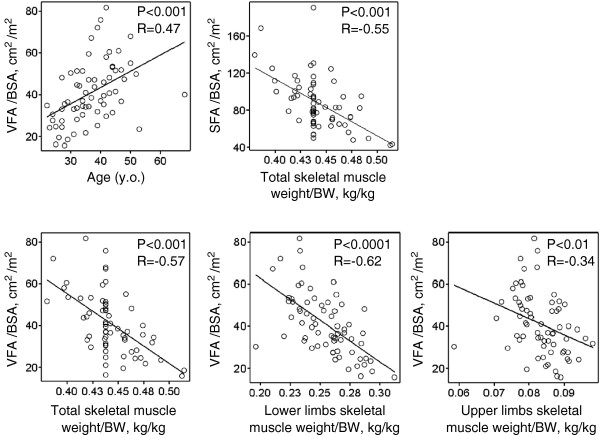
**Age is positively associated with increased VFA, while total skeletal muscle mass is negatively associated with both VFA and SFA.** Upper and lower skeletal muscle mass are negatively associated with VFA. VFA: visceral fat area, SFA: subcutaneous fat area, BSA: body surface area, BW: body weight.

The VFAI was positively associated with waist circumference, BMI, SFAI (Figure 1), and age (Figure 2), but was negatively associated with upper, lower and total skeletal muscle mass (Figure 2). The SFAI was negatively associated with total skeletal muscle mass (Figure 2). Neither SFAI nor skeletal muscle mass was associated with age (data not shown).

**Figure 3:**
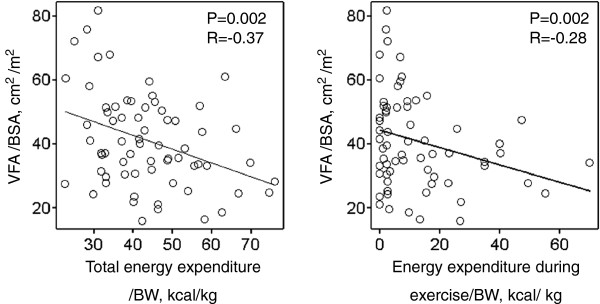
**Total energy expenditure for daily-life activity and energy expenditure during exercise are negatively associated with increased VFA.** VFA: visceral fat area, BSA: body surface area, BW: body weight.

The VFAI was negatively associated with total energy expenditure and energy expenditure during exercise (Figure 3), but there was no relationship between total caloric intake and VFAI (data not shown).

Stepwise multiple regression analysis showed that total skeletal muscle mass was a negative and age was a positive determinant of VFAI; however, total caloric intake, total energy expenditure, and energy expenditure during exercise were statistically excluded (Table 2).

**Table 2:** Multiple regression analysis for determinants of visceral fat area

**Variables**	**Coefficient**	**95% Confidence interval**	**Standardized coefficient**	***P*** **value**
Total skeletal muscle mass	−295	−404 to −187	−0.51	<0.001
Age	0.63	0.32 to 0.94	0.38	<0.001

R^2^ = 0.45, *P* < 0.001.

### VFAI is inversely associated with lower limb skeletal muscle mass

In order to clarify which part of skeletal muscle, including upper limb, lower limb, and truncal skeletal muscle, influences the volume of visceral fat, we performed stepwise multiple regression analysis. Although lower limb skeletal muscle mass was a negative and age was a positive determinant of VFA, upper limb and truncal skeletal muscle mass were statistically excluded (Table 3).

**Table 3:** Multiple regression analysis for determinants of visceral fat area

**Variables**	**Coefficient**	**95% Confidence interval**	**Standardized coefficient**	***P*** **value**
Age	0.52	0.20 to 0.84	0.31	<0.01
Lower limb muscle weight	−6.78	−9.19 to −4.36	−0.53	<0.001
Upper limb muscle weight	-	-	-	-
Truncal muscle weight	-	-	-	-

R^2^ = 0.46, *P* < 0.001.

## Discussion

The lifestyle-related factors associated with visceral fat mass has been unknown. In this study, we showed that skeletal muscle mass especially lower limb muscle mass are negatively associated with VFAI.

We showed that the VFAI is positively associated with age and negatively associated with skeletal muscle mass. VFA is positively associated with number of metabolic risk factors in the elderly [16], and skeletal muscle mass is inversely associated with age [17,18]. However, our data showed that skeletal muscle mass was not associated with age, which is supported by the evidence that muscular strength is inversely associated with the incidence of MetS, independently of age [19]. Although the age outliers might have affected the results (Additional file 1), they nevertheless indicate that the decrease in skeletal muscle mass can be prevented by physical activity.

Decreased skeletal muscle mass leads to physical inactivity [20]. Conversely, physical inactivity leads to decreased skeletal muscle mass [20]. Decreased skeletal muscle mass and strength is associated with increased morality [21,22]. Sarcopenic obesity is also associated with hypertension, independent of abdominal obesity [23]. Increasing skeletal muscle mass and strength via daily exercise may therefore prevent MetS and prolong life span.

In addition, in patients with metabolic syndrome, visceral fat accumulation is accompanied by excess lipid deposition in skeletal muscle, which may contribute to impaired glucose uptake leading to insulin resistance [24]. Improved skeletal muscle functions (including metabolic system) through exercise may contribute to the prevention of MetS [25].

The American Heart Association recommends daily exercise to prevent the accumulation of abdominal fat [1]. Although some subjects exercised in their spare time, our results showed that the association between total energy expenditure during daily activity and VFAI was stronger than the association between energy expenditure during exercise and VFAI. Because the duration of energy expenditure during exercise is relatively short, it may be insufficient for reducing VFA. Therefore, the length of continuous caloric consumption is important for reducing VFA. Enhanced energy expenditure combined with daily exercise is essential for reducing the volume of visceral fat.

Lower limb muscle mass is a determinant of VFAI. Lower limb muscle including the quadriceps forms the largest muscle mass in the body and may therefore contribute to decreased VFA to a greater extent than upper limb or truncal muscle. Lower limb muscle mass and performance in gait are also important because they are associated with reduced mobility, a poor quality of life, CVD, and death [26-28]. Increased physical activity and daily lower body exercise (e.g., brisk walking, cycling, and stair climbing) may be the most useful way to reduce visceral fat and improve mortality. Increased daily activity in young- and middle-aged men may prevent MetS and CVD by decreasing the volume of visceral fat.

In conclusion, skeletal muscle mass especially lower limb muscle mass negatively contributed to VFA in healthy men. Maintaining lower limb muscular fitness through daily exercise may therefore be a useful strategy for controlling visceral obesity and MetS.

### Consent

Informed consent was obtained from the participants for the publication of this report and any accompanying images.

## Abbreviations

MetS: Metabolic syndrome; CVD: Cardiovascular disease; VFA: Visceral fat area; SFA: Subcutaneous fat area; BMI: Body mass index; BSA: Body surface area; VFAI: Visceral fat area index; SFAI: Subcutaneous fat area index.

## Competing interests

The authors declare that they have no competing interest.

## Authors’ contributions

SY, MK, KN, TH, TIs, YU, and TIw collected data and SY analyzed the data and wrote the manuscript. KA, MA, MSh, SK, and MSa provided the suggestion for this study. All authors read and approved the final manuscript.

## Supplementary Material

Additional file 1 Age distribution of participants. Click here for file
