# Supplementary figures and images for: Association of lower limb muscle mass and energy expenditure with visceral fat mass in healthy men
Source: Diabetol Metab Syndr. 2014 Feb 26;6:27. doi: 10.1186/1758-5996-6-27 (PMC3945716; doi:10.1186/1758-5996-6-27)

## Slide 1
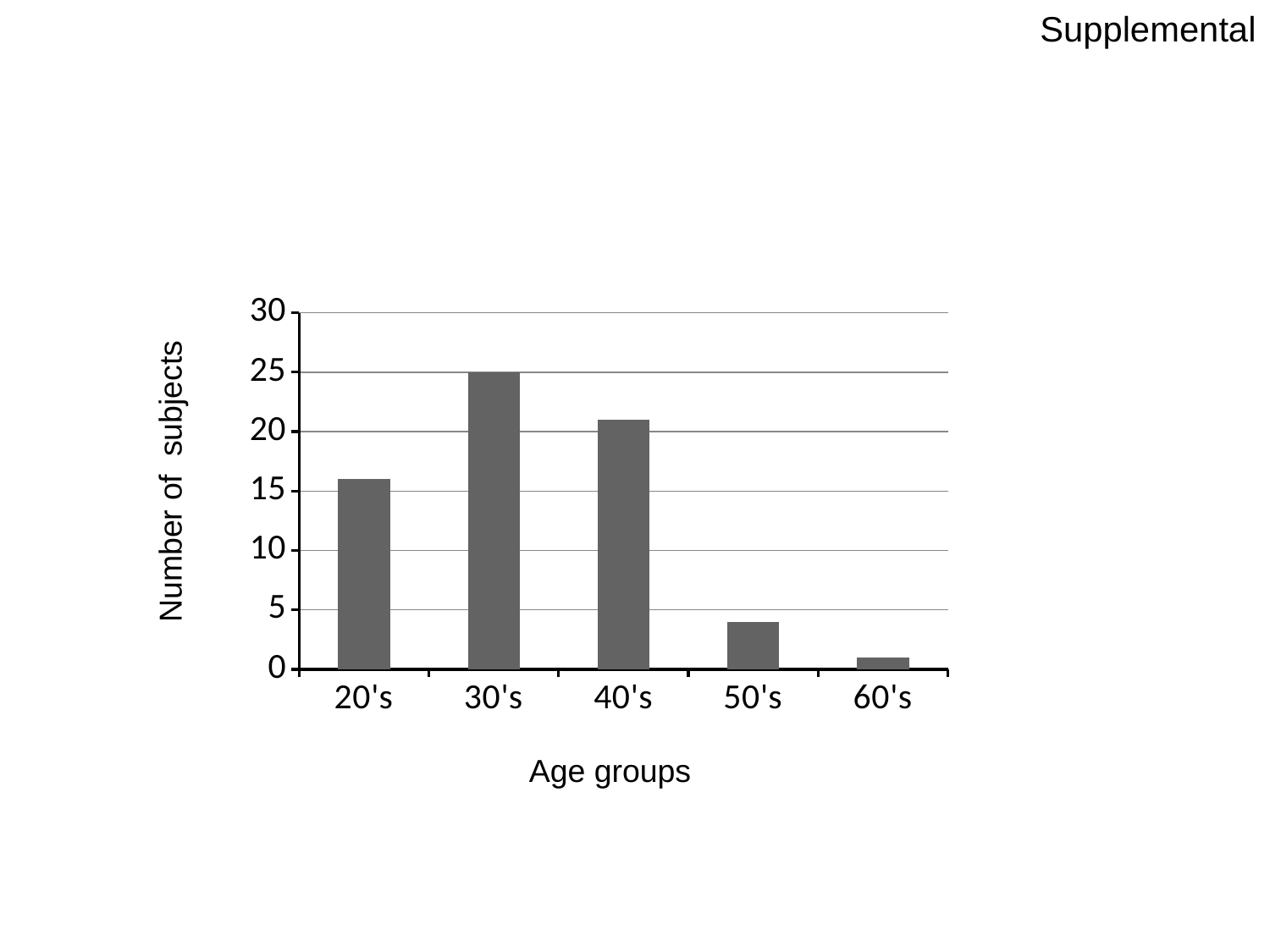

Supplemental
### Chart
| Category | |
|---|---|
| 20's | 16.0 |
| 30's | 25.0 |
| 40's | 21.0 |
| 50's | 4.0 |
| 60's | 1.0 |Number of subjects
Age groups

Supplement: Additional file 1 — Age distribution of participants. [file 1758-5996-6-27-S1.pptx]
